# Disruption of DNA Repair as an Emerging Epigenetic Mechanism Underlying Autism Spectrum Disorder

**DOI:** 10.1007/s11920-026-01684-2

**Published:** 2026-05-30

**Authors:** Benjamin Kelvington, Jaekyoon Kim, Sourav Banerjee, Hanna E. Stevens, Kim Hei-Man Chow, Ted Abel

**Affiliations:** 1 Department of Neuroscience and Pharmacology, Carver College of Medicine, University of Iowa, 169 Newton Rd, Iowa City, IA 52242 USA; 2 Iowa Neuroscience Institute, University of Iowa, Iowa City, USA; 3 Pharmacology Graduate Program, Department of Neuroscience and Pharmacology, Carver College of Medicine, University of Iowa, Iowa City, USA; 4 National Brain Research Centre, Gurgaon, India; 5 Department of Psychiatry, Carver College of Medicine, The University of Iowa, Iowa City, USA; 6 School of Life Sciences, Faculty of Science, The Chinese University of Hong Kong, Hong Kong, Hong Kong; 7 Gerald Choa Neuroscience Institute, The Chinese University of Hong Kong, Hong Kong, Hong Kong

**Keywords:** Autism Spectrum Disorder, Epigenetics, DNA Damage, DNA Repair

## Abstract

**Purpose of Review:**

To summarize recent advances in our conceptual understanding of the epigenetic mechanisms of DNA repair contributing to autism spectrum disorder (ASD).

**Recent Findings:**

Large-scale genetic studies indicate that genes involved in DNA repair contribute to some cases of ASD, and smaller scale studies have reported increased DNA damage in peripheral tissues of both individuals with ASD and their parents. ASD-associated chromatin remodelers, thought to contribute to ASD by regulating gene expression, also facilitate DNA repair. Recent evidence has provided some mechanistic insight into the function of ASD-associated genes in the epigenetic regulation of DNA repair. Importantly, the disruption of DNA repair is a leading candidate to explain the emerging connection between ASD and neurodegenerative disease and may also provide insight into sex differences in ASD.

**Summary:**

Here, we highlight recent evidence that dysfunction of the DNA repair machinery is an overlooked mechanism underlying ASD, one which requires further systematic study for the benefit of individuals with ASD and their support systems.

**Supplementary Information:**

The online version contains supplementary material available at 10.1007/s11920-026-01684-2.

## Introduction

Epigenetics refers to a diverse array of molecular mechanisms that regulate gene activity without altering DNA sequence (Table 1, Glossary of Epigenetic Terms). Research over the past decade has shed light on how epigenetics contribute to autism spectrum disorder (ASD) by focusing on the regulation of gene expression [1–4]. Large-scale genomic sequencing studies implicate genes involved in epigenetic processes that regulate gene expression including histone post-translational modification (PTM) and chromatin remodeling as top epigenetic contributors to ASD [5, 6]. For example, *FMR1*, *MECP2*, and *CHD8* are well-studied regulators of gene expression where loss of function leads to ASD-associated syndromes [7–9]. Additionally, mutations in transcription factors, regulatory regions, DNA methyltransferases, splicing factors, and environmental factors converge to disrupt the expression of genes associated with nervous system functioning [10]. These findings have led to an abundance of studies examining the impact of ASD-associated genes on transcription. However, growing evidence describing the range of functions of chromatin remodelers suggests that additional epigenetic processes are disrupted in the context of ASD with a leading candidate being DNA repair. DNA repair is not only a component of cellular aging. Neurodevelopment and neuronal activity are energy intensive processes that under typical conditions generate DNA damage as a result of multiple cellular functions, including gene transcription, DNA replication, and oxidative stress from extensive mitochondrial respiration [11, 12]. This damage creates a unique need for high-fidelity DNA repair within the nervous system. However, the mechanisms that control the optimal choice of DNA repair pathway in the brain, particularly for post-mitotic neurons with little flexibility to engage the typical high-fidelity homology-directed repair that can occur when cells are replicating DNA, are not well defined. Given this knowledge gap, the contributions of DNA repair, and the disruption of its epigenetic regulation, to ASD have been underappreciated.

**Table 1:** Glossary of terms related to epigenetics and DNA repair

Term	Definition
Acetylation	A post-translational modification where an acetyl group is added to a molecular substrate
Acetyltransferase	An enzyme that catalyzes acetylation
ADP-ribosylation	A post-translational modification where an ADP-ribose group is added to a molecular substrate
ADP-ribosylhydrolase	An enzyme that catalyzes the removal of an ADP-ribose group
Base Excision Repair (BER)	The process of repairing non-bulky DNA lesions caused by modification of nitrogenous bases
Chromatin Remodeling	The dynamic regulation of chromatin conformation that controls the ability for proteins involved in processes including transcription and DNA repair to access DNA
Coactivator	Proteins that mediate the effects of transcriptional activators often through histone acetylation
Epigenetics	Molecular mechanisms that regulate gene activity but not the underlying DNA sequence
Deacetylation	A post-translational modification where an acetyl group is removed from a molecular substrate
Demethylase	An enzyme that catalyzes the removal of methylation
Demethylation	A post-translational modification where a methyl group is removed from a molecular substrate
Deubiquitinase	An enzyme that removes ubiquitin from a molecular substrate
DNA Damage Response (DDR)	The array of molecular events occurring after the damaging of DNA in reaction to that damage
DNA Double-strand Break (DSB)	A form of DNA damage where both strands of DNA are severed
DNA Single-strand Break (SSB)	A form of DNA damage where one strand of DNA is severed
Histones	Nuclear proteins that dynamically regulate DNA, histones and DNA together form chromatin
Homologous Recombination (HR)	The process of repairing DNA damage, typically double-strand breaks, in an error-free manner by using an identical nucleic acid sequence such as a homologous chromosome or sister chromatid
Kinase	An enzyme that catalyzes phosphorylation
Long Non-coding RNA (lncRNA)	RNA molecules longer than 200 nucleotides that do not code for protein but may have other molecular functions
Methyltransferase	An enzyme that catalyzes methylation
Methylation	The deposition of a methyl group on a molecular substrate, typically DNA or histones
Mismatch Repair	The process of repairing DNA lesions produced by incorrectly paired bases
Non-homologous End Joining (NHEJ)	The error-prone process of repairing DNA damage, typically double-strand breaks, without using an identical nucleic acid sequence
Nucleosome	A unit of chromatin typically consisting of 147 base pairs of DNA wrapped around a histone octamer
Nucleotide Excision Repair (NER)	The process of repairing bulky DNA lesions caused by modification of nitrogenous bases
Phosphorylation	A post-translational modification where a phosphate group is added to a molecular substrate
Pleiotropy	The capacity for a biological molecule to affect multiple different biological pathways or phenotypic outcomes
Post-translational Modification (PTM)	Chemical modification occurring after a protein is translated that regulates its function
R-loop	A molecular structure where one strand of DNA is displaced by RNA, when unresolved can lead to DNA damage
Topoisomerase	An enzyme that catalyzes the severing of DNA strands
Ubiquitination	A post-translational modification where a ubiquitin group is added to a molecular substrate

Here, we describe the recent evidence implicating DNA repair as a function of many ASD-associated genes and contend that disruption of DNA repair is a heretofore overlooked epigenetic mechanism in the etiology of ASD. This hypothesis is supported by recent evidence from large-scale genetic studies, new revelations in the DNA repair functions of ASD-associated chromatin remodelers, and the delineation of pleiotropic roles for well-studied ASD genes in DNA repair. Although the role of DNA repair disruption in ASD and its associated challenges is just beginning to be understood, this framework may help explain the emerging connection between ASD and neurodegenerative disease as well as illuminate some mechanisms underlying sex differences in ASD.

## Human Studies Point to DNA Repair in ASD

Recent genetic studies of humans with ASD implicate DNA repair genes in ASD pathophysiology. A landmark study by Litman et al. suggests that disruption of DNA repair and chromatin remodeling pathways contributes to a specific ASD profile characterized by social/behavioral challenges [13]. Another smaller-scale study from Cunningham and colleagues implicates defects in genes involved in p53-dependent DNA repair and the Fanconi Anemia Complex as playing a role in the acute developmental regression observed in neurodevelopmental disorders including ASD [14]. Together, these high-impact studies shine a light on DNA repair in the context of ASD and suggest that DNA repair disruption may mediate a specific subset of ASD cases. Furthermore, the SFARI Gene database, the preeminent resource for ASD genetics, lists at least 104 genes that function in DNA repair (Table 2, Supplemental Table 1). This constitutes 8.2% of all genes listed, and the broad range of DNA repair functions represented by this list indicates that disruption of multiple DNA repair pathways may contribute to ASD. In addition to single genes contributing to ASD diagnoses, copy number variations (CNVs), rare genetic variations in which multiple adjacent genes are deleted or duplicated, confer a large risk for ASD and contain genes that function in DNA repair [15–18]. For example, deletion of the 16p11.2 region, among the most common genetic variations associated with ASD, results in reduced copy number of multiple DNA repair genes including *PPP4C*, *PAGR1α*, and the chromatin remodeler *INO80E* [19]. To complement genetic evidence, multiple studies reveal that both individuals with ASD and their parents have elevated DNA damage in peripheral tissues, hinting toward a reduced capacity for DNA repair [20–24]. Although this data comes from a collectively small number of individuals, when taken together with data from genetic studies, they make a convincing case for increased investigation into the roles that DNA damage and repair play in ASD.

**Table 2:** SFARI ASD risk genes with functions in DNA damage and repair

Gene	SFARI score	Role in DNA Repair
*ABL2*	3	Tyrosine kinase involved in DDR signaling, evidence not as strong as for *ABL1*
*ACTB*	1 S	Actin polymerization repositions and facilitates the repair of DSBs in heterochromatin
*ADNP*	1 S	Transcription factor that reduces DSB formation
*AHNAK*	2	Large nucleoprotein that promotes NHEJ through interactions with 53BP1
*AR*	2	Androgen receptor promotes expression of DNA repair genes
*ARID1A*	3 S	Chromatin remodeler in the SWI/SNF family that facilitates NHEJ
*ARID1B*	1 S	Chromatin remodeler in the SWI/SNF family that facilitates NHEJ
*ARID2*	2 S	Chromatin remodeler in the SWI/SNF family that promotes HR, NHEJ, and BER
*ATRX*	1	Chromatin remodeler that deposits histone variant H3.3 and facilitates sister chromatid exchange during HR
*BICRA*	2 S	Increases NHEJ through participation in the GBAF complex
*BRCA2*	2	Central regulator of HR through stabilization of RAD51.
*BTAF1*	2	RNA Polymerase II associated factor that prevents DNA damage associated with transcriptional stress
*CDKL5*	1 S	Serine/threonine kinase that promotes DNA repair possibly through interaction with PARP1
*CECR2*	2	Bromodomain protein that inhibits yH2AX activation
*CHD1*	2 S	ATP-dependent chromatin remodeler that promotes HR by regulating stability of 53BP1.
*CHD2*	1 S	ATP-dependent chromatin remodeler that promotes NHEJ through interaction with PARP1.
*CHD3*	1 S	ATP-dependent chromatin remodeler that promotes chromatin relaxation and DNA repair following PAR-dependent recruitment to sites of damage.
*CHD4*	3 S	ATP-dependent chromatin remodeler that promotes chromatin relaxation and DNA repair following PAR-dependent recruitment to sites of damage.
*CHD7*	1 S	ATP-dependent chromatin remodeler that relaxes chromatin and promotes NHEJ by regulating 53BP1 recruitment.
*CHD8*	1 S	ATP-dependent chromatin remodeler that influences DNA damage response and DNA repair gene expression through interaction with p53
*CREBBP*	1 S	Histone acetyltransferase that regulates the expression and activity of base excision repair factors
*CSNK1E*	2	Serine/threonine kinase participates in DNA repair through phosphorylation of targets including TOP2A
*CTCF*	1 S	Chromatin binding protein that promotes HR through interaction with RAD51
*CUL4B*	3 S	E3 ubiquitin ligase that facilitates DNA repair through polyubiquitination and degradation of targets including p53 and HUWE1
*CUX1*	2	Transcription factor that promotes DNA repair through transcriptional regulation of ATM and ATR
*CUX2*	2 S	Transcription factor that promotes repair of oxidative DNA damage through interaction with OGG1
*CXXC5*	3	Required for the DNA damage dependent activation of p53 and ATM
*DDX3X*	1 S	RNA helicase that promotes HR through interaction with RAD51
*DHX9*	3 S	DNA-RNA helicase that is phosphorylated by ATM and promotes HR through interaction with IGF2BP2
*DNMT3A*	1 S	*De novo* DNA methyltransferase that promotes HR through regulation of repair genes including PARP1
*DOT1L*	2 S	Methyltransferase whose methylation of RAP80 is required for recruitment of BRCA1
*DYRK1A*	1 S	Dual specificity kinase that increases DNA damage in Down Syndrome through interactions with repair proteins 53BP1 and RNF169
*EPC2*	2	Polycomb protein member of NuA4 complex that promotes HR
*EP300*	1 S	Transcriptional co-activator that stabilizes NBS1 in response to ATM activation
*EP400*	2	ATPase and member of NuA4 complex with multiple roles in DNA repair including facilitating HR through interaction with RAD51
*FAN1*	2	Required for interstrand cross-link repair with both endonuclease and exonuclease activity.
*FMR1*	1 S	Facilitates transcription coupled HR through RNA methylation and the resolution of R loops
*FRG1*	2	Promotes DNA repair through transcriptional regulation of base excision repair genes
*HDAC4*	2 S	Histone deacetylase recruited to DNA damage sites that regulates HR through H4K120 acetylation and interacts with 53BP1
*HDAC8*	S	Histone deacetylase that regulates DNA damage through expression of repair factors including MRE11
*HDLBP*	1	High density lipoprotein binding protein that promotes DNA repair through interactions with RAD51 and BRCA1
*HERC2*	S	E3 ubiquitin ligase that recruits repair genes to sites of DNA damage
*HMGN1*	2	Nucleosome binding protein associated with transcriptionally active chromatin that promotes repair by increasing chromatin accessibility
*HNRNPU*	1 S	Member of ribonucleoprotein complexes that promotes repair through interactions with both NHEJ and R-loop complexes.
*IKZF1*	3	Promotes HR through interaction with USP7
*ILF2*	2	Transcription factor that in complex with ILF3 promotes NHEJ and mediates activity of lncRNA involved in DNA damage response
*KDM2B*	1	Histone lysine demethylase whose repression leads to accumulation of NHEJ factors.
*KDM5C*	1	Histone lysine demethylase that promotes DNA repair during embryonic development
*KDM6A*	2	X-linked histone lysine demethylase that regulates expression of DNA repair genes
*KDM6B*	1	Histone lysine demethylase that regulates the expression of the DNA repair gene MGMT
*KMT2C*	1 S	Histone lysine methyltransferase that regulates expression of HR factors
*KMT5B*	1	Histone lysine methyltransferase that targets H4K20 and regulates NHEJ
*MACROD2*	2	Removes ADP-ribose from mono-ADP-ribosylated proteins and impacts DNA repair through interaction with PARP1
*MBD1*	2	Promotes genome stability during replication stress through interaction with PARP1 at replication fork
*MBD4*	2	Methyl-CpG binding protein with DNA glycosylase activity involved in mismatch repair
*MCM6*	2	Component of MCM DNA helicase complex that facilitates DNA repair resulting from replication stress
*MCPH1*	2	Multiple roles in DNA damage response including promoting ATR signaling through interaction with TOPBP1
*MECP2*	1 S	Promotes DNA repair through interaction with PARP1
*MED23*	2	Decreases DNA repair by inhibiting expression and recruitment of nucleotide excision repair factors
*NIPBL*	1 S	Recruits the cohesin complex to sites of DNA damage
*NR4A2*	1	Activity-induced transcription factor that promotes DNA repair through interactions with PARP1 and DNA-PK
*NSD2*	2 S	Methyltransferase that facilitates the interaction between PTEN and 53BP1
*POLA2*	2	Part of DNA polymerase alpha complex involved in initiation of DNA replication that promotes both HR and NHEJ
*POLR2A*	3 S	Subunit of RNA polymerase II whose ubiquitylation and degradation promotes DNA-PK dependent NHEJ
*POLR3A*	3 S	Catalytic subunit of RNA polymerase III that functions in HR by synthesizing RNA-DNA hybrids that protect 3’ overhangs
*PRKDC*	2	Catalytic subunit of DNA-PK, a central regulator of NHEJ through interactions with Ku70/80.
*PRPF19*	3	Ubiquitin ligase with multiple roles in DNA repair including through recognition of RPA and promotion of ATR signaling
*PRR12*	1 S	Proline-rich protein that localizes to sites of DNA damage along with NIPBL and the cohesin complex
*PSMD6*	1	Member of 26 S proteosome recruited to the nucleus in response to DNA damage
*PTEN*	1 S	Phosphatase that negatively regulates PI3K signaling with SUMOylation dependent nuclear localization and DNA repair function
*PUF60*	3 S	Splicing factor that regulates the splicing and expression of core DNA repair genes
*RAD21*	S	Component of cohesin complex involved in DNA repair and cohesion of sister chromatids during mitosis
*SATB1*	1 S	Stimulates base excision repair through interaction with OGG1
*SETBP1*	1	Binds the SET nuclear oncogene and inhibits p53 function leading to damage accumulation
*SIN3B*	2 S	Scaffolding protein and transcriptional co-repressor recruited to the site of DNA damage that recruits MDC1 and promotes alternative NHEJ
*SMAD4*	2	Member of SMAD family of signaling factors that promotes DSB repair
*SMARCA1*	1	Member of ATP-dependent chromatin remodeling complex essential for multiple DNA damage response pathways
*SMARCA2*	1 S	Member of ATP-dependent chromatin remodeling complex that promotes HR mediated by RAD51
*SMARCA4*	1	Member of ATP-dependent chromatin remodeling complex that promotes HR mediated by RAD51
*SON*	1 S	RNA splicing cofactor that regulates expression of multiple DNA repair genes
*SPTAN1*	3	Spectrin protein recruited to sites of DNA damage that promotes repair of interstrand cross-links
*SRCAP*	1	Member of chromatin remodeling complex required for end resection during HR
*SRSF1*	3 S	Promotes interstrand crosslink repair and suppresses R-loop formation through interaction with FANCD2.
*STAG1*	S	Member of cohesin complex that regulates DNA damage and S phase checkpoint
*SUPT16H*	2 S	Member of FACT complex that controls nucleosome assembly required for DNA repair.
*TAOK1*	1 S	Serine/threonine kinase that promotes HR through phosphorylation of USP7 and stabilization of RAD51
*TET2*	2	Involved in DNA demethylation and protects against DNA damage through interaction with p53
*TET3*	S	Links 5-methylcytosine oxidation to base excision repair in the process of DNA demethylation
*TLK2*	1 S	Serine/threonine kinase that is recruited to sites of DNA damage through interaction with PCNA and promotes NHEJ
*TOP2B*	2	Topoisomerase that produces double-strand DNA breaks.
*TOP3B*	2	Topoisomerase that leads to transient breaks of a single strand of DNA
*TRIP12*	1 S	E3 ubiquitin ligase that regulates DNA damage responses through ubiquitination of RNF168 and USP7
*TRRAP*	1 S	Part of NuA4 complex that promotes repair through recruitment of histone acetyltransferases
*TTI1*	S	Member of Triple T complex that regulates DNA damage response through interaction with ATM
*UBE3A*	1 S	E3 ubiquitin ligase that regulates degradation of RAD51
*UIMC1*	2	Interacts with BRCA1 and binds ubiquitinated histones to promote HR.
*USP7*	2 S	Deubiquitinates DNA repair proteins including Rad18
*USP9X*	1 S	Deubiquitinase that regulates HR through interactions with BRCA1 and RAD51
*VCP*	3	Interacts with ubiquitinated proteins and facilitates the recruitment of 53BP1
*VDR*	2	Vitamin D receptor that promotes nucleotide excision repair through interaction with XPC
*WAC*	1 S	Member of ubiquitination complex targeting H2B that impacts transcriptional responses to DNA damage through interaction with p53
*XPC*	S	DNA damage sensing and DNA binding activity is central to nucleotide excision repair
*XRCC6*	3	Helicase that promotes NHEJ through recruitment of DNA-PK
*ZNF865*	1	Zinc finger transcription factor that regulates expression of DNA repair genes

## Mechanistic Impacts of ASD-risk Genes on DNA Repair

**Fig. 1:**
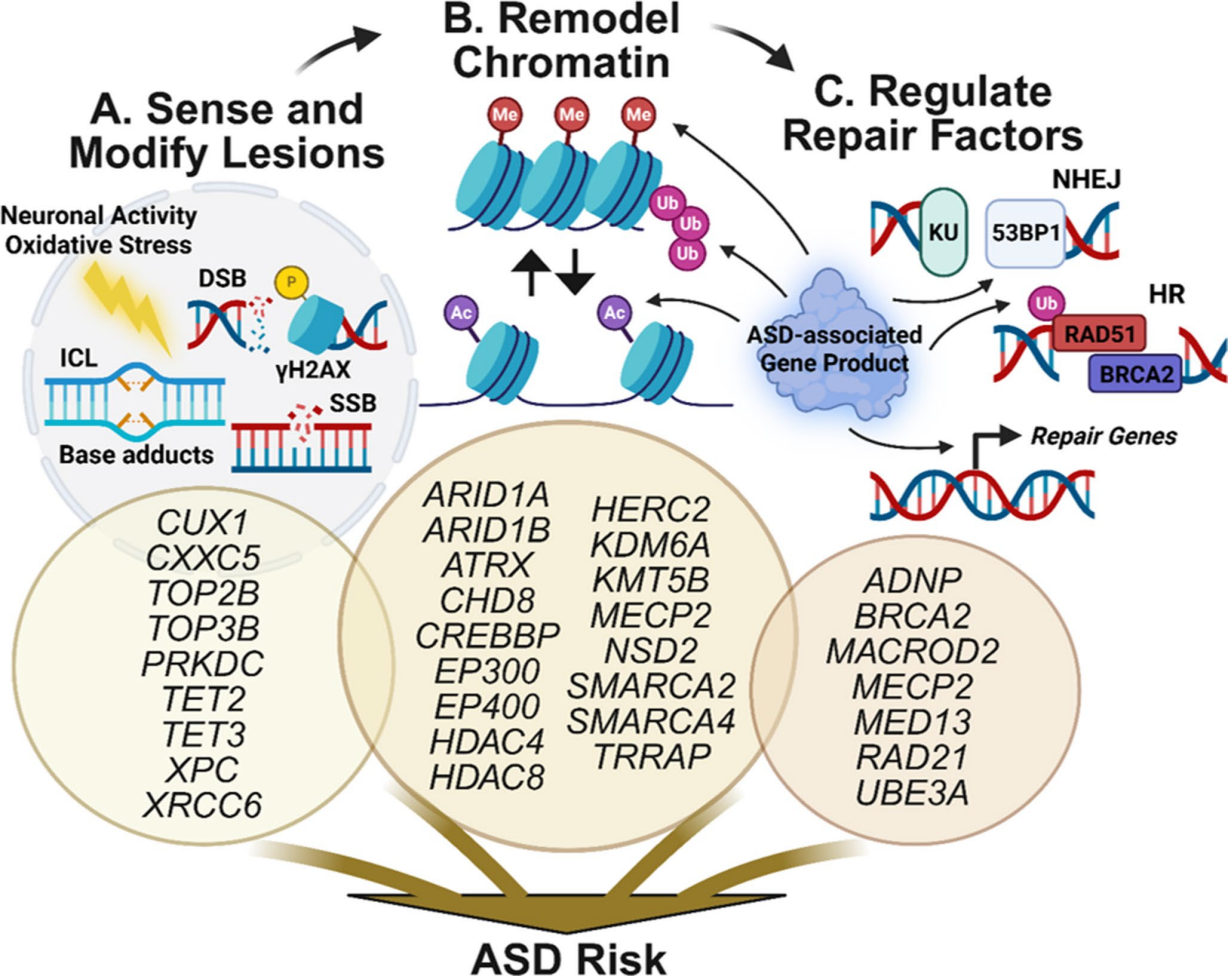
Epigenetic contributions to DNA repair by ASD-risk genes. The products of ASD-risk genes are involved in three interconnected events critical to maintaining genomic stability. **A** Sensing and modifying DNA lesions: During both neurodevelopment and mature neuronal activity, high levels of DNA damage are generated by multiple processes including transcription and oxidative stress. ASD-risk genes are implicated in sensing these lesions and initiating the DNA damage response via modulating the lesion site structure and dynamics. **B** Chromatin remodeling: ASD-associated chromatin remodelers mediate changes in histone post-translational profile and nucleosome architecture to facilitate DNA repair. These processes include histone post-translational modifications (e.g., methylation [Me], ubiquitination [Ub], acetylation [Ac]), histone eviction or degradation, and the exchange of histone variants. **C** Regulating repair factors: ASD-risk genes play a role in recruiting and regulating, both directly through post-translational modification and indirectly through transcriptional control of repair factors, the engagement of context-specific DNA repair pathways. These pathways include non-homologous end joining (NHEJ) and homologous recombination (HR) for DNA double-strand breaks (DSBs) and the resolution of interstrand crosslinks (ICLs) and single-strand breaks (SSBs). This framework underscores the multifaceted role of ASD-risk genes in regulating DNA repair through epigenetic mechanisms, linking neurodevelopmental processes to the maintenance of genomic integrity through DNA repair

Although the evidence linking DNA repair to ASD is just beginning to emerge, the function of ASD-risk genes points to their role in the epigenetic regulation of DNA repair, which must be finely tuned to respond to damage in a lesion-specific and context-specific manner [25, 26]. While the mechanisms by which disruption of DNA repair leads to the challenges associated with ASD are unknown, many ASD-risk genes implicated in the epigenetic regulation of multiple DNA repair pathways have been investigated in other contexts [27, 28]. Here, we discuss the potential mechanistic role for ASD-risk genes in epigenetically regulating DNA repair by sensing and modifying DNA lesions, remodeling chromatin, and broadly modulating repair factors, processes which are interconnected and overlapping (Fig. 1).

### ASD Gene Products Sense and Modify DNA Lesions to Facilitate Repair

The DNA damage response (DDR) begins when DNA lesions form and kinases are activated to initiate lesion-specific and context-dependent signaling pathways [29] (Fig. 1A). Several SFARI ASD risk genes play a direct role in the deposition and sensing of DNA damage, including *PRKDC* and *XRCC6*, coding for the DNA-PK catalytic subunit and KU70, respectively. These proteins control the sensing of DNA double-strand breaks (DSBs) and their repair through the non-homologous end joining (NHEJ) pathway [30]. Additionally, hyperactivity of ATM, a kinase central to DDR regulation, has recently become a target of interest in ASD [31, 32]. Although loss of ATM is commonly associated with cognitive deficits and neurodegeneration in ataxia telangiectasia, sustained ATM activity can detrimentally promote an unresolved DDR and the accumulation of repair intermediates [33]. ATM expression is induced in both mice exposed to valproic acid, an environmental contributor to ASD risk, and mice modeling Rett syndrome, ultimately resulting in GABAergic dysfunction [31]. ATM expression and activation are also regulated by ASD-risk genes *CUX1* and *CXXC5*, suggesting that regulation of DDR signaling at multiple levels is impacted in ASD [34, 35]. In addition to sensing DNA damage, gene products of SFARI ASD-risk genes play important roles in processing DNA lesions to facilitate efficient repair. For example, *TOP2B* and *TOP3B*, topoisomerases involved in the generation of DSBs and single-strand breaks (SSBs), respectively, introduce temporary breaks that are necessary for effective base excision repair (BER) [36–38]. Similarly, *TET2* and *TET3*, encoding enzymes responsible for the elimination of methylated cytosines from DNA, create a more accessible chromatin state for BER [39, 40]. Additionally, *XPC* encodes a sensor for bulky DNA adducts that initiates nucleotide excision repair (NER) [41, 42]. When taken together, these data highlight how disruption of genes involved in DDR at the DNA level contribute to ASD.

### ASD Gene Products Remodel Chromatin to Promote DNA Repair

Beyond events at the DNA level, chromatin remodeling through histone PTMs is a fundamental epigenetic component of DNA repair [29] (Fig. 1B). Several genes encoding histone variants (*H1-4*,* H2BC11*,* H3-3B*,* H4C11*,* H4C3*,* and H4C5*) are on the SFARI list, suggesting that changes in nucleosome composition are likely to play a role in ASD by altering chromatin. Acetylation, methylation, phosphorylation, ubiquitination, and polyADP-ribosylation of these histones are among the PTMs known to regulate DNA repair among other impacts [43, 44]. These modifications are controlled by multiple families of chromatin remodeling complexes, whose components include the products of numerous ASD-risk genes. For instance, *SMARCA2* and *SMARCA4* are members of the SWI/SNF family of chromatin remodelers that are crucial for nucleosome organization after DNA lesion sensing and promote the homologous recombination (HR) pathway of DSB repair [45]. Within chromatin remodeling complexes are regulators of histone PTMs such as *HDAC4*, a SFARI gene that regulates HR through deacetylation of histone variant H2B at lysine 120 [46]. Dynamic changes in histone acetylation are indeed a core event of DDR and heavily implicated in the pathogenesis of ASD [4, 44, 47]. A recent landmark study by Pollina et al. demonstrates how ASD-associated histone acetyltransferases function within chromatin remodeling complexes to repair neuronal activity-dependent DSBs [48]. NPAS4, whose expression is induced rapidly by neuronal activity, recruits the NuA4 chromatin remodeling complex containing the histone acetyltransferase *TRRAP*, a SFARI gene associated with a neurodevelopmental syndrome [49–51], to the site of DNA damage, ultimately mediating repair. Deletion of *TIP60*, another histone acetyltransferase within the NuA4 complex associated with a neurodevelopmental syndrome [52], reproduces DNA damage and gene expression profiles observed with loss of *NPAS4* [48]. Although the participation of additional ASD-associated gene products in this process remains unknown, *NR4A2*, another gene rapidly induced by neuronal activity, and in which mutations cause a developmental syndrome associated with ASD [53], also functions to promote DNA repair [54, 55]. Chromatin remodeling through histone PTMs is also intimately related to the recruitment of other DNA repair factors, for instance the ASD-associated DNA repair gene product HDLBP [56]. Together, these studies provide mechanistic insight into how histone acetylation remodels chromatin to promote DNA repair and how disruption of this process may contribute to ASD.

In addition to histone acetylation, other PTMs both play a role in fine-tuning DDR and are implicated in ASD. For example, ubiquitination of histones influences DNA repair by regulating chromatin architecture at sites adjacent to DSBs [29]. Reductions in H2AK119ub1 result in delayed DNA repair during neurogenesis [57], and this reduction can result from disruption of SFARI gene *ASXL3*, a component of the Polycomb repressive deubiquitinase complex [58]. Other deubiquitinases such as USP7 and USP9X also both mediate DNA repair and are implicated in ASD [59–62]. Multiple histone PTMs can also interact to influence DNA repair, and these interactions can be mediated by SFARI ASD-risk genes. For instance, recent evidence reported by Tang et al. demonstrates that the recruitment of BRCA1, a core protein promoting HR, is dependent upon both histone methylation by *DOT1L* and recognition of ubiquitinated histones by RAP80 (encoded by *UIMC1*), both ASD-risk genes [63]. ASD-risk gene products also remodel chromatin in the service of DNA repair by facilitating histone variant exchange: *ATRX* deposits the histone variant H3.3 to facilitate HR, in addition to its role in sister chromatid exchange [64]. The studies collectively suggest a central role for ASD-risk genes in promoting DNA repair through chromatin remodeling.

### ASD Gene Products Regulate DDR Factors

Beyond regulating chromatin through histone PTMs, recent research has identified roles for ASD-risk gene products as post-translational modifiers of key DDR proteins themselves (Fig. 1C). For example, in addition to histone modification, ubiquitination serves as a recruitment platform for repair machinery that dictates repair pathway choice. For instance, ubiquitination by *UBE3A* was recently shown to regulate core HR component RAD51 [65]. ADP-ribosylation provides an additional layer of protein regulation and is mediated by the HR gene PARP1 [29]. Interestingly, *MACROD2*, encoding a mono-ADP-ribosylhydrolase, an eraser of ADP-ribosylation and negative regulator of PARP1, is also an ASD-risk gene [66]. In addition to regulating DDR factors, ASD-risk genes can be DNA repair factors or members of DNA repair complexes themselves. Notably, *BRCA2* is responsible for the loading of HR mediator RAD51 onto SSBs [67]. Additionally, *RAD21* is a member of the cohesin complex and directly participates in DNA repair through the cohesion of sister chromatids [68]. These studies indicate that regulation of DNA repair machinery, as well as the DNA repair machinery itself, is susceptible to disruption by mutations in ASD-risk genes.

In addition to directly regulating DDR factors, ASD-associated genes are well-known for their broad impacts on gene transcription, which may in turn interfere with the capacity for DNA repair [35, 36]. Notably, a recent report from Wan et al. revealed that the transcriptional impact of ASD-associated chromatin remodelers *ADNP*, *KDM6B*, *CHD2*, and *MED13* converges on changing the expression of DNA repair genes, suggesting that the transcriptional impact of loss of chromatin remodelers associated with autism reduces the capacity for DNA repair [69]. Moreover, multiple studies have revealed that the expression of DDR components is tightly regulated by the balanced activities of ASD-associated histone lysine methyltransferases and demethylases [70–73]. The apparent transcriptional effects of histone methylation on DNA repair may also be mediated by other protein functions. For example, two recent reports from Morales et al. demonstrate that loss-of-function mutations in the methyl-CpG binding protein 2 (*MECP2*), causative of Rett Syndrome, are related to a transcriptome-wide changes impacting DNA damage and cellular senescence, but may also be attributable to its regulatory interaction with PARP1 [74, 75]. Together these studies indicate that the transcriptional consequences of ASD-associated mutations chromatin remodelers may be due to both regulation of DDR-related transcriptional programs and emerging direct interactions with the DDR machinery.

To fully understand the mechanisms underlying DNA damage in the context of ASD, it is essential to adopt a broader perspective on well-studied high-confidence autism genes, particularly those with emerging pleiotropic roles in DNA repair. For example, the product of *FMR1*, well known for its role in suppressing activity-dependent translation at the synapse, has recently been shown to promote transcription-coupled DNA repair by regulating methyl-5-cytosine levels of mRNA [76]. *PTEN*, a tumor suppressor known for negatively regulating AKT signaling, also plays a role in DNA repair [77]. PTEN mutations leading to nuclear exclusion and diminished PTEN-dependent DNA repair are associated with ASD [78]. Additionally, the roles of ASD-associated genes in DNA repair may become apparent only when studying them in particular cellular contexts or during particular stages of the cell cycle. For instance, chromatin regulators ADNP, CHD8, CHD2, POGZ, and KMT5B, which are associated with ASD, localize to microtubules during nuclear envelope breakdown in mitosis, where they regulate DNA damage and cell cycle progression [79]. Careful molecular dissection will be required to assign the relative contribution of different molecular functions to ASD-related challenges, but these data suggest that ASD risk genes are highly pleiotropic and that the DNA repair-related functions of many ASD-associated genes may remain to be discovered. Moreover, DNA repair is likely to play a role in ASD even in the absence of a single known genetic cause. For example, decreased expression of DNA repair genes causes ASD-relevant phenotypes in the BTBR mouse, a model without specific known risk gene variants [80, 81]. Collectively, these findings suggest that autism researchers should consider the impact of DNA damage and repair processes, even when such mechanisms are not immediately apparent.

### Potential Links Between the Disruption of DNA Repair and ASD-Associated Impairments

As the genetic and mechanistic links between DNA repair and ASD begin to emerge, more work is needed to clarify the neurobiological consequences of DNA repair dysfunction that could produce the challenges experienced by individuals with ASD. Disruption in DNA repair may impact nervous system development or mature functioning through multiple potential pathways. One such pathway involves a disruption of the adaptive role that DNA breaks play in the brain [82]. Although typically considered damaging, DNA breaks are known to play multiple roles that are essential for proper nervous system function, and disruption of these strictly regulated processes is likely to have detrimental consequences [82]. For instance, a recent report from Jovasevic et al. details how DNA damage in a subset of neurons leads to extranuclear release of DNA and histones, which drives persistent innate immune signaling that is indispensable for long-term memory [83, 84]. The induction of DNA damage in response to learning in a restricted cellular ensemble, as well as the reliance on inflammatory signaling also associated with neural dysfunction, suggests that this learning process may also be susceptible to perturbation of underlying DNA repair pathways. Additionally, DNA breaks induced by *TOP2B* are required for the induction of gene expression both in response to experience and in long genes critical for neuronal function [85–89], single-strand breaks play a critical role at neuronal enhancers [90, 91], oxidative DNA damage regulates the proliferation of neuronal progenitors [92, 93], and areas of recurrent DNA breaks in neuronal genes may contribute to the generation of neuronal diversity [94, 95]. Together, these findings indicate that multiple fundamental pathways critical for nervous system function are reliant upon high-fidelity DNA repair and thus are susceptible to DNA repair dysfunction. Such vulnerabilities may ultimately contribute to the development of ASD.

Beyond disrupting the adaptive role of DNA breaks, DNA repair infidelity is likely to produce detrimental consequences due to the accumulation of excess DNA damage and DNA repair intermediates. For example, although reactive oxygen species during development regulate the differentiation of neural progenitors, excess reactive oxygen species also contribute to cellular toxicity and death of neural progenitors [96]. Thus, reduced DNA repair capacity during critical developmental windows including neurogenesis may result in accumulation of excess oxidative damage, which is likely to disrupt neurodevelopment and cause functional deficits later in life. Indeed, stress associated with hyperproliferation during neurogenesis in a model of ASD with macrocephaly disrupts the expression of ASD-associated genes [97]. Relatedly, the accumulation of DNA damage during neurodevelopment is likely to produce somatic mutations that persist to alter mature neuronal function [98], and somatic mutations concentrated in ASD-associated genes contribute a significant risk for ASD [99, 100]. For example, *ADNP*, an ASD-associated gene encoding a chromatin remodeler, is highly susceptible to somatic mutations, which can result in neuronal dysfunction and death [101]. Additionally, the disruption of synaptic genes, which tend to be long and are prone to transcription-induced DNA breaks, may affect mature neuronal function [88, 94]. Consequently, neurons harboring excessive DNA damage may either adopt a senescence-associated secretory phenotype, characterized by both the loss of neuronal functions and the promotion of neuroinflammation, or alternatively undergo apoptosis [102]. For example, *CDKL5*, an ASD-related gene, is associated with both DNA damage and apoptosis when disrupted [103]. These examples represent multiple plausible explanations for the functional connection between DNA repair and the challenges associated with ASD, but the exact pathways remain to be defined.

## DNA Repair Connects ASD to Neurodegenerative Disorders

Accumulating evidence suggests that early neurodevelopmental events influence neurodegenerative outcomes [104], an observation supported by recent studies indicating a significantly elevated risk of neurodegenerative conditions, including Parkinson’s Disease and Alzheimer’s Disease, among individuals with ASD [105–110]. The accumulation of DNA damage is a major contributor to neurodegenerative disease and is intricately linked to chromatin dysregulation, suggesting that disruption of DNA repair genes in ASD may increase susceptibility to neurodegeneration [12, 111–113]. Although the precise epigenetic mechanisms connecting DNA repair infidelity to both neurodevelopmental and neurodegenerative disorders remain unclear, valuable insights can be gleaned from the shared genetic pathways implicated in these conditions. For example, mutations in *NR4A2*, an ASD risk gene, have also been implicated in early onset dystonia-parkinsonism [114, 115]. Although the specific contributions of *NR4A2*‘s transcriptional versus DNA repair functions to these conditions remain unclear, this connection suggests that the DNA repair functions of activity-induced genes may represent a shared mechanism underlying both neurodevelopmental and neurodegenerative disorders. Additionally, the link between FXS and its associated neurodegenerative condition Fragile X-associated tremor/ataxia syndrome is mediated by the number of CGG repeats in the *FMR1* locus [7]. Repeat length is regulated by a cycle of DNA demethylation, R-loop formation, and DNA repair, highlighting how epigenetic regulation of DNA repair at the *FMR1* locus may influence the balance between neurodevelopmental and neurodegenerative outcomes [116]. Moreover, mutations in ATM, the central regulator of DDR signaling mutations and an emerging target in ASD, result in the neurodegenerative disease Ataxia-Telangiectasia characterized by progressive cerebellar atrophy [32]. Beyond single-gene mutations, developmental syndromes resulting from CNVs including Down Syndrome and 22q11.2 deletion syndrome are linked to early onset neurodegeneration, leading to Alzheimer’s Disease and Parkinson’s disease, respectively [117, 118]. While the role of DNA damage in the developmental consequences of these syndromes and the specific genes involved remain insufficiently characterized, existing evidence indicates that dose imbalance of *DYRK1A* drives DNA damage accumulation in Down syndrome [119]. The cumulative burden of excess DNA damage over a lifetime is a well-established driver of neurodegeneration, but more work is needed to elucidate how early-life DNA damage contributes to neurodevelopmental disorders including ASD.

An emerging mechanism influencing DNA repair, which may link ASD and neurodegeneration, is epigenetic regulation by long non-coding RNAs (lncRNAs). lncRNAs are known to be dysregulated in ASD, and their roles in DNA repair and neurodegeneration are beginning to be uncovered [120, 121]. For instance, a recent study identified a novel lncRNA, *Discn*, which is induced by DNA damage and facilitates DNA repair by recognizing single stranded DNA fragments [122]. Loss of *Discn* leads to the accumulation of DNA damage and triggers an inflammatory response mediated by type-I interferon signaling. Similarly, a recent study identified Brain Specific DNA-damage Related lncRNA1 (*BS-DRL1*), which influences neurodegeneration through its interaction with HMGB1, a non-histone chromatin remodeler involved in DNA repair [123]. *BS-DRL1* promotes the recruitment of HMGB1 to damaged DNA, and loss of *BS-DRL1* results in extensive DNA damage and progressive Purkinje neuron degeneration. Although future research will be needed to fully elucidate the roles of lncRNAs in DNA repair, because of their known dysregulation in ASD, their disruption represents a promising mechanism connecting ASD and neurodegenerative diseases. Overall, the epigenetic regulation of DNA repair is a compelling candidate to explain the link between ASD and neurodegeneration with the potential to inform both lifelong support strategies for individuals with ASD across the lifespan and early interventions for neurodegenerative disorders.

## DNA Repair may Influence Sex Bias of ASD

Considering DNA repair as a potential mediator of sex differences in ASD may offer a unifying framework linking genetic burden, epigenetic regulation, and neurodevelopmental vulnerability to ASD-associated challenges. One of the most well-documented findings in ASD research is its strong male bias [124, 125]. Despite extensive efforts, the biological mechanisms driving this sex difference remain incompletely understood. Notably, sex differences in DNA damage and repair are well documented in cancer biology [126–128]. However, whether sex-dependent regulation of DNA repair contributes to increased ASD risk in males requires further investigation.

Sex chromosome–linked genomic mechanisms provide a compelling framework for sex differences in DNA repair capacity relevant to ASD. Many X-linked genes encode proteins involved in transcriptional regulation, chromatin remodeling, and DNA damage responses [129, 130]. Notably, KDM6A, an X-linked histone lysine demethylase that escapes X chromosome inactivation, has emerged as a key regulator of DNA damage responses through modulation of the p53 and retinoblastoma (Rb) pathways [126, 131]. KDM6A directly regulates canonical p53 targets, including *CDKN1A* (p21), and coordinates transcriptional programs that restrain cell-cycle progression and maintain genomic stability [132, 133]. Supporting this idea, p21 expression is consistently higher in female tissues across species, and female cells exhibit greater p21 induction following DNA damage [134, 135]. Although p53 itself is autosomal, many of its downstream targets are X-linked [136], suggesting that the X chromosome may bias sex-dependent DNA repair and stress responses, and disruption of p53 signaling produces more pronounced behavioral and synaptic deficits in male mice [137]. Moreover, X chromosome genes are enriched among ASD-associated DNA repair genes with 12 genes in total (*AR*,* ATRX*,* CDKL5*,* CUL4B*,* DDX3X*,* FMR1*,* HDAC8*,* KDM5C*,* KDM6A*,* MECP2*,* SMARCA1* and *USP9X*) localized to the X (Table 2), supporting a model in which X-linked regulation of DNA repair pathways contributes to sex-specific neurodevelopmental vulnerability in ASD.

Sex steroid hormones offer an additional framework for understanding sex differences in DNA repair capacity relevant to ASD by exerting sex-dependent effects on DNA damage responses and repair pathways. While much of the mechanistic insight comes from cancer biology, a body of work shows that estrogens enhance DNA repair by promoting NHEJ and improving mismatch and nucleotide excision repair, while reducing oxidative DNA damage through activation of PI3K/Akt–Nrf2 signaling and induction of brain-derived neurotrophic factor (BDNF), leading to increased expression of repair enzymes such as APE1 [138, 139]. Furthermore, estrogen signaling regulates key components of the DNA damage response, including ATM, ATR, CHK1, p53, and BRCA1/2, and influences repair pathway choice via estrogen receptor–dependent complexes [140–142]. Similarly, androgens directly regulate multiple components of DNA double-strand break repair, and the androgen receptor, an X-linked ASD-risk gene, interacts with a broad network of DNA repair–related genes [142, 143]. Although brain-focused studies remain limited, excessive androgen exposure during early development disrupts social and cognitive behaviors in a male-specific manner [144], suggesting heightened vulnerability to hormone-mediated genomic stress. It is also likely that complex interplay between sex hormones modulates neurodevelopment and DNA repair processes. This idea is supported by findings that the chromatin remodeler TIP60 interacts differentially with androgen, estrogen, and progesterone receptors through both direct acetylation of receptors and as a transcriptional coactivator, suggesting that the impact of ASD-associated chromatin remodelers on transcription and DNA repair may be modulated by sex-specific steroid hormone profiles [145]. Together, these findings support a model in which sex steroid hormones shape DNA repair capacity during neurodevelopment, potentially contributing to sex-biased susceptibility to ASD through differential regulation of genomic stability and stress responses.

## Conclusions

In this review we synthesize recent genetic evidence, as well as mechanistic analyses of ASD-associated genes, that implicates DNA damage repair in the etiology of ASD. This evidence reveals that multiple overlapping mechanisms, including epigenetic regulation by chromatin remodelers, may mediate the connection between DNA repair and ASD. This perspective not only provides insights into the molecular underpinnings of ASD but may also help explain some of its other notable characteristics, such as a pronounced sex bias and an emerging association with neurodegenerative diseases. Further research in humans including analysis of peripheral tissue samples, i*n vivo* imaging, and post-mortem investigations will be essential to define the extent and localization of DNA damage accumulation in ASD. Additionally, mechanistic investigation of diverse model systems will be necessary to elucidate the precise molecular machinery and functional consequences connecting ASD-associated repair genes to the challenges faced by individuals with ASD. These investigations will inform the extent to which DNA damage contributes to the challenges associated with ASD and whether it represents a viable therapeutic target.

While many drugs, particularly chemotherapeutics, are designed to increase DNA damage, relatively few therapeutic strategies aim to reduce DNA damage or enhance DNA repair. Promising candidates include enoxacin, which promotes DNA repair through the upregulation of repair-promoting miRNAs and is currently in clinical trials for ALS [146, 147], and FDA-approved antioxidant agents such as amifostine and edavarone, which reduce oxidative DNA damage and improve ASD-relevant phenotypes by mitigating reactive oxygen species [148, 149]. Although clinical trials on the effects of antioxidants in ASD have produced mixed results, these studies assess relatively short periods of intervention [150–153]. It may be relevant for clinicians to take a lifespan approach and consider strategies to minimize oxidative stress and the resulting DNA damage that might be particularly impactful during critical developmental windows or accumulate over a lifetime to influence neurodegenerative outcomes. Reducing oxidative stress serves as an initial approach to limit DNA damage while more targeted therapeutics, informed by the results of mechanistic studies on DNA repair in ASD, are developed. Increased knowledge of the role of DNA repair in ASD also serves as a point of caution for the development treatment strategies that induce DNA damage, including AAV-based or CRISPR-based approaches, as recent evidence indicates that AAV-mediated toxicity in neural progenitors arises from inhibition of DDR factors [154]. Overall, our review argues for an increased appreciation of the roles of DNA damage and repair in ASD and suggests that fully understanding the underlying mechanisms can inform the development of therapeutic supports with the potential to improve the lives of people with ASD.

## Supplementary Information

Supplementary Material 1. Table S1: SFARI ASD risk genes with functions in DNA damage and repair extended information.

## Data Availability

No datasets were generated or analysed during the current study.

## Key References

- Litman A, Sauerwald N, Green Snyder L, Foss-Feig J, Park CY, Hao Y, et al. Decomposition of phenotypic heterogeneity in autism reveals underlying genetic programs. Nat Genet. 2025;57(7):1611–9.
  - ○ This analysis of genetic and phenotypic data delineates four clinically relevant classes of autism each with unique underlying molecular programs. Epigenetic processes connected to DNA repair are implicated across classes, and regulation of DNA repair is denoted as a biological process impacted in the social/behavioral class, which is characterized clinically by limited social communication.
- Cunningham JL, Frankovich J, Dubin RA, Pedrosa E, Baykara RN, Schlenk NC, et al. Ultrarare Variants in DNA Damage Repair Genes in Pediatric Acute-Onset Neuropsychiatric Syndrome or Acute Behavioral Regression in Neurodevelopmental Disorders. Dev Neurosci. 2025;47(4):231-50.
  - ○ This genetic analysis of 17 cases of children with either acute onset of psychiatric symptoms or acute behavioral regression associated with autism or another NDD reveals involvement of a subset of genes functioning in p53-dependent DNA repair and the Fanconi Anemia DNA repair complex.
- Pollina EA, Gilliam DT, Landau AT, Lin C, Pajarillo N, Davis CP, et al. A NPAS4-NuA4 complex couples synaptic activity to DNA repair. Nature. 2023;614(7949):732-41.
  - ○ This paper elucidates the mechanism coupling activity-dependent DNA breaks to rapid DNA repair in neurons through an interaction between NPAS4 and the NuA4 chromatin remodeling complex which contains the autism associated histone acetyltransferase TRRAP. Deletion of TIP60, another histone acetyltransferase member of the NuA4 complex associated with a neurodevelopmental syndrome, results in DNA damage and gene expression profiles similar to those observed with loss of NPAS4.
- Tang H, Lu YF, Zeng R, Liu C, Shu Y, Wu Y, et al. DOT1L-mediated RAP80 methylation promotes BRCA1 recruitment to elicit DNA repair. Proc Natl Acad Sci U S A. 2024;121(35):e2320804121.
  - ○ This mechanistic study elucidates how the products of ASD-risk genes *DOT1L* and *UIMC1* interact to mediate DNA repair through the post-translational modification of DNA repair factors and binding of ubiquitinated histones.
- Wan L, Yang G, Yan Z. Identification of a molecular network regulated by multiple ASD high risk genes. Hum Mol Genet. 2024;33(13):1176–85.
  - ○ This analysis of chromatin immunoprecipitation with sequencing experiments finds that ASD-associated transcriptional regulators are enriched at the promoters of genes involved in DNA repair.
- Jovasevic V, Wood EM, Cicvaric A, Zhang H, Petrovic Z, Carboncino A, et al. Formation of memory assemblies through the DNA-sensing TLR9 pathway. Nature. 2024;628(8006):145–53.
  - ○ This paper identifies that sustained DNA damage in a subset of hippocampal neurons activates the DNA-sensing TLR9 pathway, a process that is required for memory, highlighting how DNA damage and repair drive unique learning-dependent processes in different cellular ensembles.

## Declarations

### Language Statement

Opinions on the preferred language for referring to individuals with ASD vary throughout the autism community. We acknowledge these differences and use person-first language in this manuscript.
