# Supplementary material for: Disruption of DNA Repair as an Emerging Epigenetic Mechanism Underlying Autism Spectrum Disorder
Source: Curr Psychiatry Rep. 2026 May 30;28(1):33. doi: 10.1007/s11920-026-01684-2 (PMC13222229; doi:10.1007/s11920-026-01684-2)
Supplement: Supplementary file 1 — Supplementary Material 1. Table S1: SFARI ASD risk genes with functions in DNA damage and repair extended information. [file 11920_2026_1684_MOESM1_ESM.docx]

**Supplemental Table 1. SFARI ASD risk genes with functions in DNA damage and repair extended information.**

| **Gene** | **Full Name** | **PMID(s)** |
| --- | --- | --- |
| *ABL2* | ABL proto-oncogene 2, non-receptor tyrosine kinase | 20841568 |
| *ACTB* | Actin beta | 31481797 |
| *ADNP* | Activity-dependent neuroprotector homeobox | 37525242 |
| *AHNAK* | AHNAKnucleoprotein | 15177040, 33961796 |
| *AR* | Androgen receptor | 41058763 |
| *ARID1A* | AT-rich interaction domain 1A | 24788099 |
| *ARID1B* | AT-rich interaction domain 1B | 24788099 |
| *ARID2* | AT-rich interaction domain 2 | 28238438, 28381560, 35017665 |
| *ATRX* | alpha thalassemia/mental retardation syndrome X-linked | 30263950 |
| *BICRA* | BRD4 interacting chromatin remodeling complex associated protein | 39394449 |
| *BRCA2* | breast cancer 2, early onset | 28976962 |
| *BTAF1* | bromodomain containing 4 | 32905765 |
| *CDKL5* | cyclin-dependent kinase-like 5 | 32002787, 41005313 |
| *CECR2* | CECR2, histone acetyl-lysine reader | 22699752 |
| *CHD1* | chromodomain helicase DNA binding protein 1 | 28383660 |
| *CHD2* | chromodomain helicase DNA binding protein 2 | 26895424 |
| *CHD3* | chromodomain helicase DNA binding protein 3 | 29733391 |
| *CHD4* | chromodomain helicase DNA binding protein 4 | 29733391 |
| *CHD7* | chromodomain helicase DNA binding protein 7 | 33188175 |
| *CHD8* | chromodomain helicase DNA binding protein 8 | 19151705 |
| *CREBBP* | CREB binding protein | 22198154, 31504229 |
| *CSNK1E* | casein kinase 1 epsilon | 19043076 |
| *CTCF* | CCCTC-binding factor | 28560323, 28973861 |
| *CUL4B* | cullin 4B | 24452595, 25883150 |
| *CUX1* | cut like homeobox 1 | 22319212 |
| *CUX2* | cut like homeobox 2 | 26221032 |
| *CXXC5* | CXXC finger protein 5 | 19557330 |
| *DDX3X* | DEAD (Asp-Glu-Ala-Asp) box helicase 3, X-linked | 38323009 |
| *DHX9* | DExH-box helicase 9 | 40716747, 41100254 |
| *DNMT3A* | DNA (cytosine-5-)-methyltransferase 3 alpha | 34215619 |
| *DOT1L* | DOT1 like histone lysine methyltransferase | 39172790 |
| *DYRK1A* | Dual-specificity tyrosine-(Y)-phosphorylation regulated kinase 1A | 37451904 |
| *EPC2* | Enhancer of polycomb homolog 2 (Drosophila) | 28884217 |
| *EP300* | E1A binding protein p300 | 20471956 |
| *EP400* | E1A binding protein p400 | 23266955 |
| *FAN1* | FANCD2/FANCI-associated nuclease 1 | 25430771 |
| *FMR1* | fragile X messenger ribonucleoprotein 1 | 35290126 |
| *FRG1* | FSHD region gene 1 | 39169067 |
| *HDAC4* | histone deacetylase 4 | 12668657, 38874468 |
| *HDAC8* | histone deacetylase 8 | 39317961 |
| *HDLBP* | high density lipoprotein binding protein | 33941620 |
| *HERC2* | HECT and RLD domain containing E3 ubiquitin protein ligase 2 | 20023648 |
| *HMGN1* | high mobility group nucleosome binding domain 1 | 12660172 |
| *HNRNPU* | heterogeneous nuclear ribonucleoprotein U | 36943867 |
| *IKZF1* | IKAROS family zinc finger 1 | 35414773 |
| *ILF2* | Interleukin enhancer binding factor 2 | 21969602 |
| *KDM2B* | lysine demethylase 2B | 26237645 |
| *KDM5C* | lysine demethylase 5C | 32211412 |
| *KDM6A* | lysine demethylase 6A | 41501085 |
| *KDM6B* | Lysine (K)-specific demethylase 6B | 35648484 |
| *KMT2C* | Lysine (K)-specific methyltransferase 2C | 30665945 |
| *KMT5B* | lysine methyltransferase 5B | 34007043 |
| *MACROD2* | MACRO domain containing 2 | 29880585 |
| *MBD1* | methyl-CpG binding domain protein 1 | 37949945 |
| *MBD4* | methyl-CpG binding domain protein 4 | 31476572 |
| *MCM6* | minichromosome maintenance complex component 6 | 34370039 |
| *MCPH1* | microcephalin 1 | 25301947 |
| *MECP2* | Methyl CpG binding protein 2 | 41005313 |
| *MED23* | mediator complex subunit 23 | 28834744 |
| *NIPBL* | Nipped-B homolog (Drosophila) | 28167679 |
| *NR4A2* | nuclear receptor subfamily 4 group A member 2 | 21979916 |
| *NSD2* | nuclear receptor binding SET domain protein 2 | 31217297 |
| *POLA2* | DNA polymerase alpha 2, accessory subunit | 32549188 |
| *POLR2A* | RNA polymerase II subunit A | 31048545 |
| *POLR3A* | RNA polymerase III subunit A | 33626331 |
| *PRKDC* | protein kinase, DNA-activated, catalytic polypeptide | 24680878 |
| *PRPF19* | pre-mRNA processing factor 19 | 24332808 |
| *PRR12* | proline rich 12 | 39742660 |
| *PSMD6* | proteasome 26S subunit, non-ATPase 6 | 24987841 |
| *PTEN* | phosphatase and tensin homolog (mutated in multiple advanced cancers 1) | 23888040 |
| *PUF60* | poly(U) binding splicing factor 60 | 34582091, 40655003 |
| *RAD21* | RAD21cohesin complex component | 20711430 |
| *SATB1* | SATB homeobox 1 | 27590341 |
| *SETBP1* | SET binding protein 1 | 34193871 |
| *SIN3B* | SIN3 transcription regulator family member B | 37314748 |
| *SMAD4* | SMAD family member 4 | 25893305 |
| *SMARCA1* | SNF2 related chromatin remodeling ATPase 1 | 25486562 |
| *SMARCA2* | SWI/SNF related, matrix associated, actin dependent regulator of chromatin, subfamily a, member 2 | 39082357 |
| *SMARCA4* | SWI/SNF related, matrix associated, actin dependent regulator of chromatin, subfamily a, member 4 | 39082357 |
| *SON* | SONDNA binding protein | 21504830 |
| *SPTAN1* | spectrin alpha, non-erythrocytic 1 | 12571280 |
| *SRCAP* | Snf2 related CREBBP activator protein | 25176633 |
| *SRSF1* | serine and arginine rich splicing factor 1 | 38165804 |
| *STAG1* | stromal antigen 1 | 24324008 |
| *SUPT16H* | SPT16 homolog, facilitates chromatin remodeling subunit | 33104782 |
| *TAOK1* | TAO kinase 1 | 40106350 |
| *TET2* | Tet methylcytosine dioxygenase 2 | 30390073 |
| *TET3* | tet methylcytosine dioxygenase 3 | 25915473 |
| *TLK2* | tousled-like kinase 2 | 39727191, 39844055 |
| *TOP2B* | DNA topoisomerase II beta | 37939182 |
| *TOP3B* | Topoisomerase (DNA) III beta | 33378676 |
| *TRIP12* | Thyroid hormone receptor interactor 12 | 22884692, 27800609 |
| *TRRAP* | transformation/transcription domain associated protein | 16382133 |
| *TTI1* | TELO2 interacting protein 2 | 40514657 |
| *UBE3A* | ubiquitin protein ligase E3A | 40087295 |
| *UIMC1* | ubiquitin interaction motif containing 1 | 39172790 |
| *USP7* | Ubiquitin specific peptidase 7 (herpes virus-associated) | 25961918 |
| *USP9X* | ubiquitin specific peptidase 9 X-linked | 31512408, 31964704 |
| *VCP* | valosin containing protein | 22120668 |
| *VDR* | vitamin D receptor | 33524369 |
| *WAC* | WW domain containing adaptor with coiled-coil | 33868474 |
| *XPC* | xeroderma pigmentosum, complementation group C | 12949838 |
| *XRCC6* | X-ray repair cross complementing 6 | 11719239 |
| *ZNF865* | zinc finger protein 865 | 40667339 |
